# Role of aberrant PI3K pathway activation in gallbladder tumorigenesis

**DOI:** 10.18632/oncotarget.1808

**Published:** 2014-03-10

**Authors:** Andrea Lunardi, Kaitlyn A. Webster, Antonella Papa, Bhavik Padmani, John G. Clohessy, Roderick T. Bronson, Pier Paolo Pandolfi

**Affiliations:** ^1^ Cancer Research Institute, Beth Israel Deaconess Cancer Center, Department of Medicine and Pathology, Beth Israel Deaconess Medical Center, Harvard Medical School, Boston, MA 02215, USA; ^2^ Preclinical Murine Pharmacogenetics Facility, Beth Israel Deaconess Medical Center, Harvard Medical School, Boston, MA, USA; ^3^ The Dana Farber/Harvard Comprehensive Cancer Center, Boston MA, USA

**Keywords:** PI3K, PTEN, gallbladder tumorigenesis, mouse model

## Abstract

The PI3K/AKT pathway governs a plethora of cellular processes, including cell growth, proliferation, and metabolism, in response to growth factors and cytokines. By acting as a unique lipid phosphatase converting phosphatidylinositol-3,4,5,-trisphosphate (PIP3) to phosphatidylinositol-4,5,-bisphosphate (PIP2), phosphatase and tensin homolog (PTEN) acts as the major cellular suppressor of PI3K signaling and AKT activation. Recently, PI3K mutations and loss/mutation of PTEN have been characterized in human gallbladder tumors; whether aberrant PTEN/PI3K pathway plays a causal role in gallbladder carcinogenesis, however, remains unknown. Herein we show that in mice, deregulation of PI3K/AKT signaling is sufficient to transform gallbladder epithelial cells and trigger fully penetrant, highly proliferative gallbladder tumors characterized by high levels of phospho-AKT. Histopathologically, these mouse tumors faithfully resemble human adenomatous gallbladder lesions. The identification of PI3K pathway deregulation as both an early event in the neoplastic transformation of the gallbladder epithelium and a main mechanism of tumor growth in *Pten* heterozygous and *Pten* mutant mouse models provides a new framework for studying *in vivo* the efficacy of target therapies directed against the PI3K pathway, as advanced metastatic tumors are often addicted to “trunkular” mutations.

## INTRODUCTION

The phosphoinositide 3-kinase (PI3K)/AKT/mTOR network is one of the most important and well-characterized signaling pathways involved in the regulation of cell growth, proliferation and survival in response to the presence of growth factors or specific hormones such as insulin [1, 2]. Upon activation of receptor tyrosine kinases (RTKs), PI3K is recruited in the plasma membrane to transform phosphatidylinositol-4,5,-bisphosphate (PI-4,5-P2) to phosphatidylinositol-3,4,5,-trisphosphate (PI-3,4,5-P3), which, in turn, is recognized and bound by proteins carrying the pleckstrin homology (PH) domains [2, 3]. A nodal member among the PH-proteins is the serine-threonine kinase AKT. Once activated through phosphorylation of threonine 308 and serine 473 by PDK1 and mTOR complex 2 (mTORC2), respectively, AKT may phosphorylate a large number of target proteins and by either inhibiting or activating them, regulate cell metabolism and growth (GSK3, mTORC1, AS160, TSC2, PRAS40), proliferation (Wee1, p21, p27Kip1), and apoptosis (XIAP, BAD, MDM2, Caspase 9, FoxO1) [4]. Activation of PI3K signaling by somatic mutations in the *PIK3CA* gene has been frequently described in glioblastoma, breast, endometrial, colorectal, and hepatocellular cancers, while amplifications of the *PIK3CA* gene are common in lung, cervical, ovarian, and gastric cancers [5].

By turning PI-3,4,5-P3 to PI-4,5-P2, the lipid phosphatase PTEN is the most important negative regulator of PI3K signaling activation, and one of the most frequently affected genes in human cancer [6, 7]. Dysfunctional levels of PTEN have been reported in the 50-80% of endometrial carcinoma, glioblastoma and prostate cancer, and in 30-50% of breast, colon and lung tumors, while germline mutations of *PTEN* have been described in a group of autosomal dominant syndromes collectively referred to as the PTEN hamartoma tumor syndromes (PHTS) [8].

In summary, over-activation of PI3K/AKT signaling is an extremely frequent event in human cancer that may impinge on many different aspects of tumorigenesis, such as cellular proliferation, resistance to apoptosis, angiogenesis, and metastasis [9].

Recently, PI3K signaling deregulation as a consequence of *PIK3CA* mutations or *PTEN* loss has been described in respectively 8% and 50% of human gallbladder carcinoma (GBC) [10-13]. GBC is the most common tumor of the biliary tract and one of the most frequent cancers of the gastrointestinal apparatus. Although relatively rare, GBC is extremely lethal with less than 5% of patients surviving beyond 5 years as most GBCs are diagnosed at an advanced stage, when chemotherapy has a limited impact and surgical resection is no longer resolutive [14]. Notably, different studies have described a correlation between the presence of polypoid lesions of the gallbladder (PLG), their size (larger polyps are more likely to contain foci of invasive cancer) and the risk of developing GBC, in turn suggesting that PLGs may evolve in GBC [15]. Since gallbladder polyps are easily detectable by ultrasonography as non-shadowing, fixed, solid masses projecting into the lumen of the gallbladder [15], this fast, cost-effective and, most importantly, painless and non-invasive type of examination might pave the way for targeted preventive therapies based on ultrasonography screening. The reliability of ultrasonography in the diagnosing of PLG as non-neoplastic or neoplastic is extremely high for lesions with diameters larger than 2 centimeters, but falls short for less extensive lesions.

Importantly, a better understanding of the molecular mechanisms responsible for the neoplastic transformation of the gallbladder mucosal epithelium and initiation of GBC may provide important insights for the identification of effective biomarkers and targeted therapies aimed at preventing the lethal progression of GBC.

## RESULTS

### *Pten^+/−^* mice develop gallbladder tumors

The PI3K/PTEN pathway was recently implicated in human gallbladder carcinoma as a consequence of PI3K mutations [10, 12] or PTEN loss [13]. Indeed, all the mutations described for the *PIK3CA* gene are well-known “hot spot” sites driving the constitutive activation of PI3K function [16]. In order to study whether PI3K/AKT hyperactivity results in the neoplastic transformation of the gallbladder epithelium, we took advantage of the *Pten* knock-out mouse model previously generated in our laboratory [17]. Although homozygous *Pten* knock-out mice are embryonically lethal, heterozygous *Pten* littermates (*Pten^+/−^*) are viable and fertile, yet highly tumor prone as consequence of the PTEN haploinsufficent regulation of the oncogenic PI3K/AKT signaling pathway [6].

**Figure 1:**
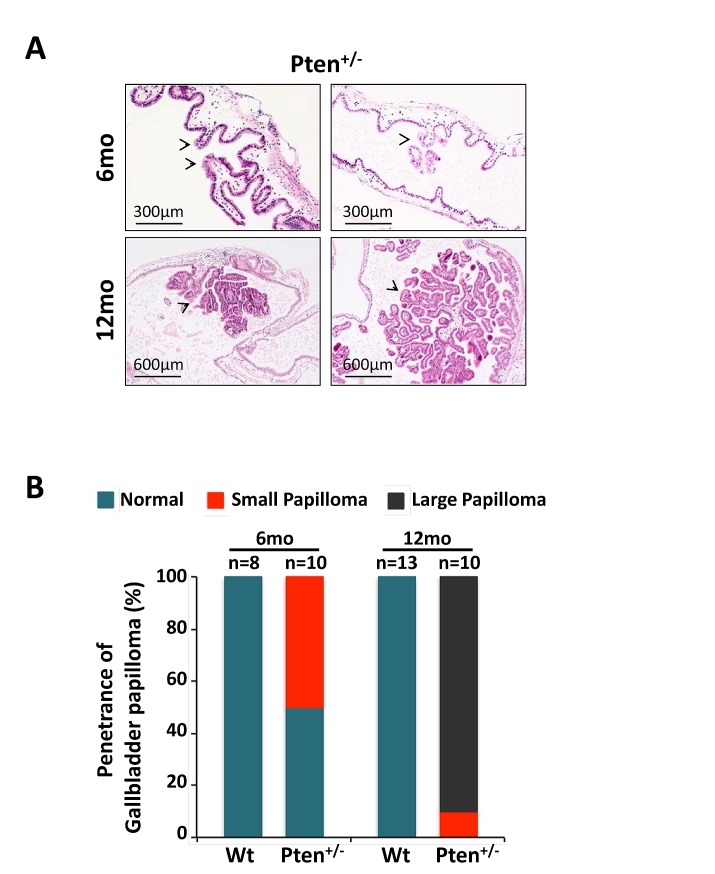
Haploinsufficient tumor suppressive function of *Pten* in gallbladder epithelial cells A) H&E staining showing micro, small, large and very large papillomas (arrowheads) in 6- and 12-month-old *Pten^+/−^* mice, respectively. (B) Penetrance of gallbladder papilloma in 6- and 12-month-old wild type and *Pten^+/−^* mice.

Two different cohorts of C57BL/6 *Pten^+/−^* mice were generated and sacrificed respectively at 6 and 12 months of age (n=10 per cohort). After euthanasia, gallbladders were extracted, fixed in 4% PFA in PBS, and embedded in paraffin for histopathological and histochemical analysis. Age-matched wild type littermates (6-month-old, n=8; 12-month-old, n=13) served as controls. In line with our hypothesis, histopathological studies on H&E sections revealed the presence of micro and small papillomas characterized by cellular pleiomorphism in 50% (5 out of 10) of 6 month-old *Pten^+/−^* mice (Fig. 1A, upper panel), while the gallbladder epithelium was normal in all age-matched wild type mice (0 out of 8) (Fig. 1B; Fig. S1). Strikingly, large papilloma lesions were found in 90% (9 out of 10) of 12-month-old *Pten^+/−^* mice, while none of the age-matched wild type mice showed signs of abnormalities in their gallbladder (0 out of 13) (Fig. 1A, lower panel; 1B; Fig. S1). Importantly, *Pten^+/−^* papillomas at 12 months of age were characterized by the papillary proliferation of uniform small epithelial cells arranged in single layers, and by the presence of a narrow stalk, devoid of invasive epithelial cells, that closely resembled human neoplastic adenomas [18].

Benign polyps of the human gallbladder are generally distinguished as non-neoplastic (e.g. cholesterol aggregates, inflammatory polyps, or adenomyomas), or neoplastic (e.g. adenomas, or leiomyomas) lesions. The most common benign neoplastic lesion of the gallbladder is the adenoma, a glandular tumor composed of cells resembling biliary tract epithelium, generally classified into papillary and non-papillary types according to histology. Importantly, neoplastic adenomatous polyps are thought to represent a form of premalignant lesion with the potential to progress to GBC [15, 19].

### Dysregulated PI3K signaling drives gallbladder epithelial cells transformation and tumor growth

**Figure 2:**
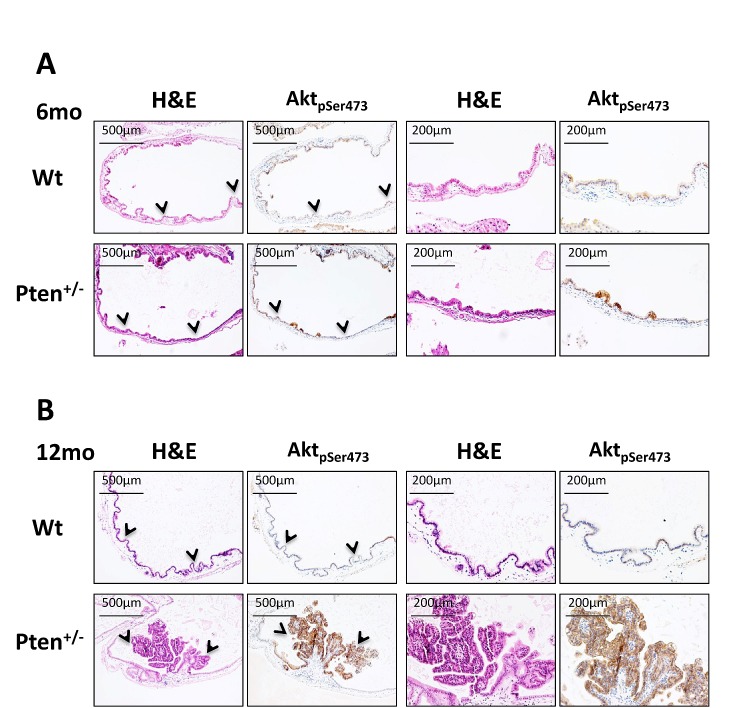
Akt hyperactivation in *Pten^+/−^* gallbladder tumors (A) H&E and pSer473Akt staining on serial sections of gallbladder collected from 6 month-old, and (B) 12 month-old wild type and *Pten^+/−^* mice. Arrowheads in the first and second panels indicate the region of tissue magnified in the third and forth panels.

To investigate whether *Pten* heterozygosity in the gallbladder epithelium was triggering PI3K activation, we next stained serial sections of gallbladder with H&E and phosphoS473-Akt. As shown in Figure 2A, micro-papillomas characterizing the results from 6-month-old *Pten^+/−^* gallbladder were clearly positive for pS473Akt, evidence of the role that PI3K signaling plays during the first steps of neoplastic cell transformation. Importantly, as shown in Fig. 2B, large papillary neoplastic polyps in 12-month-old *Pten^+/−^* gallbladders were found to be still characterized by high levels of pS473Akt, while the epithelium of both 6- and 12-month-old wild type mice showed no signs of PI3K/Akt activation (Fig. 2A-B, upper panels; Fig. 4C).

Overall these results suggest a fundamental role of PI3K/AKT in the gallbladder tumorigenesis not only by promoting the initial neoplastic transformation of the gallbladder epithelial cells, but also by sustaining the growth of early lesions to large papillomas, strongly supporting the hypothesis of the addiction of these tumors to PI3K/AKT hyperactivity.

### *Pten^+/−^* gallbladder adenomas are highly proliferative

**Figure 3:**
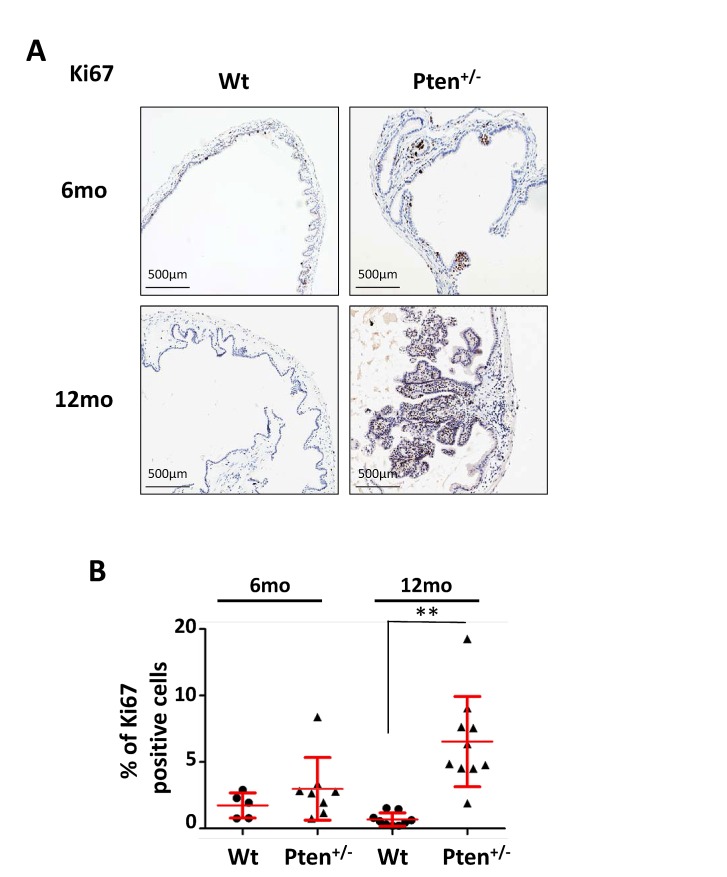
High frequency of Ki67 positive cells in *Pten^+/−^* gallbladder adenomas (A) Ki67 staining of gallbladder epithelium of 6 and 12 month-old wild type and *Pten^+/−^* mice. (B) Percentage of Ki67 positive gallbladder epithelial cells in the cohorts of mice described in (A). Red bars in the dot-plot represent mean value ± s.d.. Data were analyzed using unpaired t-test. *p*Values<0.01 were considered statistically significant (**p<0.01).

Although PI3K/AKT activity is preferentially linked to cell growth and survival, AKT oncogenic function has also been related to cell proliferation [4]. To analyze the proliferative index of papillomas, we stained 6- and 12-month-old wild type and *Pten^+/−^* gallbladders for the proliferation marker Ki67. As shown in Figure 3A (upper panels), Ki67 positive nuclei were particularly enriched in small papilloma lesions, although the difference between wild type and *Pten^+/−^* gallbladder was not statistically significant (Fig. 3B). On the contrary, the proliferation index level in the 12 month-old *Pten^+/−^* papillomas was 5 times higher than in wild type littermates (Fig. 3A lower panels; 3B; *p*=0.00018).

**Figure 4:**
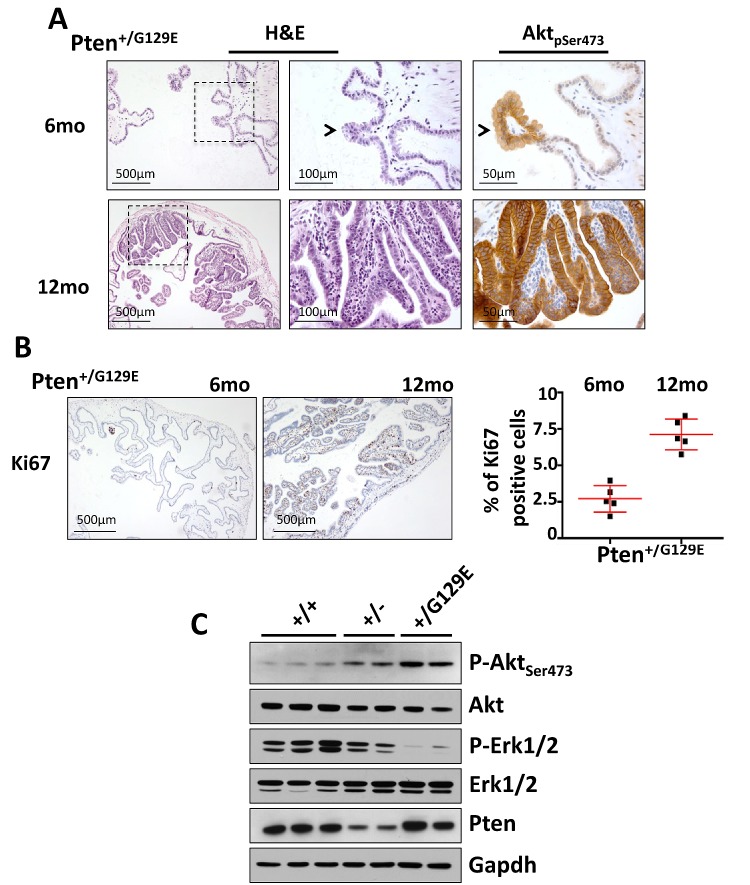
Gallbladder tumors in *Pten^G129E^* mutant mice (A) H&E staining of gallbladder extracted from 6 and 12 month-old *Pten^+/G129E^* mice (left panels). Magnifications of the region of tissue indicated by the dashed square are shown in the middle panels. pSer473Akt staining was done on serial sections (right panels). Arrowheads indicate the same lesion in the two panels. (B) Ki67 staining of gallbladder epithelium of 6- and 12-month-old *Pten^+/G129E^* mice. Percentage of Ki67 positive gallbladder epithelial cells in the cohorts of mice described in (B) is shown in the graph. Red bars in the dot-plot represent mean value ± s.d.. (C), Western blot analysis of 12-month old gallbladders collected from wild type (n=3), *Pten^+/−^* (n=2), and *Pten^+/G129E^* (n=2) mice.

### Lipid phosphatase dead *Pten^G129E^* mutant mice develop aggressive gallbladder tumors

The onco-suppressive role of PTEN was initially attributed to the inhibition of PI3K activity through its lipid phosphatase function, however, other important roles of PTEN have been discovered in the control of genomic stability, and the cell cycle progression over the past few years [20]. To understand if the neoplastic transformation of the gallbladder epithelium in the *Pten^+/−^* mice was exquisitely dependent on the hyperactivation of the PI3K/AKT pathway, or some combination of the multiple tumor suppressive function of PTEN, we analyzed a specific cohort of knock-in mice carrying the *Pten*-G129E mutant allele in heterozygosity [21]. The G129E missense mutation located in the catalytic pocket of PTEN is known to abolish its lipid phosphatase activity specifically, without affecting any of the protein phosphatase function or phosphatase independent functions of this tumor suppressor [22].

We found that in our cohort of *Pten^+/G129E^* mice, 100% of samples (n=5) developed papillomas by 6 months of age (Fig. 4A, left and middle upper panels). These papillomas had progressed to very large polyps in all the 12-month-old *Pten^+/G129E^* mice analyzed (n=5) (Fig. 4A, left and middle lower panels). As expected, strong pSer473-Akt staining and a high proliferative index (Ki67) were identified by immunohistochemistry in the lesions of all the mice studied (Fig. 4A, right upper and lower panels; 4B). Interestingly, *Pten^+/G129E^* mice displayed early polyps (at 6 months of age) and more aggressive gallbladder papillomas (Grade 2 at 12 months of age) than *Pten^+/−^* mice (Grade 1 at 12 months of age). Western blot analysis with total lysates of gallbladders from 12-month old wild type (n=3), *Pten^+/−^* (n=2), and *Pten^+/G129E^* (n=2) mice showed comparable levels of Pten in both wild type and *Pten^+/G129E^* lysates, while the reduction in Pten levels was evident in the *Pten^+/−^* samples (Fig. 4C). Accordingly, we found higher levels of phospho-Ser473Akt in *Pten^+/−^* and *Pten^+/G129E^* compared to wild type samples, with the *Pten^+/G129E^* samples presenting the highest signal. Finally, in line with the previously characterized cross-talk between PI3K and MAPK signaling [23-25], we found a robust reduction in the level of phospho-Erk in both the *Pten^+/−^* and *Pten^+/G129E^* compared to wild type samples, with the *Pten^+/G129E^* samples presenting the lowest signal (Fig. 4C).

Collectively, these data confirm the dominant negative function of mutant PtenG129E over the wild type Pten protein in PI3K signaling inhibition, as recently described by our group [21], and further support the thesis of PI3K/AKT signaling deregulation as a main oncogenic driver in the gallbladder tumorigenesis.

### *In vivo* detection of gallbladder tumors in *Pten^+/−^* mice

**Figure 5:**
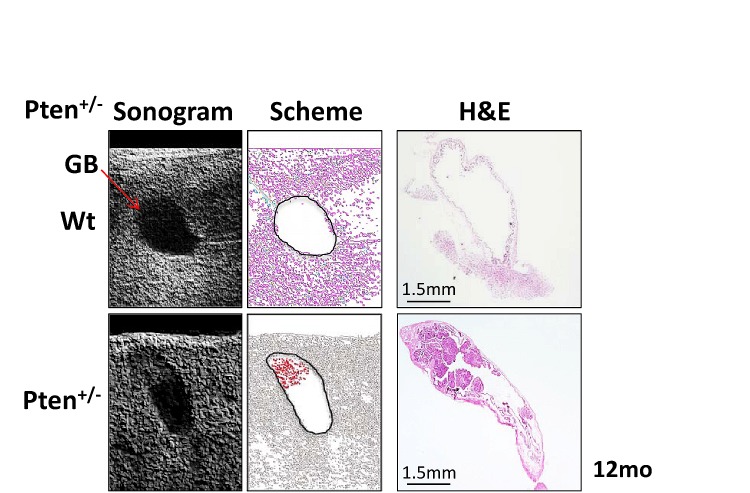
Ultrasonogram analysis Left panels: gallbladder (arrow) in 12 month-old wild type (upper) and *Pten^+/−^* (lower) mice. Middle panels: a scheme of the ultrasonogram acquisition shows two black circles bounding the gallbladder of wild type (upper) and *Pten^+/−^* (lower) mice and in red, the dense mass identified by ultrasonography in the apical region of the *Pten^+/−^* gallbladder. Right panels: H&E of the wild type (upper) and *Pten^+/−^* (lower) gallbladders analyzed by ultrasonography showing the large papilloma in the apical part of the *Pten^+/−^* gallbladder.

Gallbladder polyps in humans are generally diagnosed by ultrasonography examination of the abdomen [14, 15]. In order to determine whether large papillomas in 12 month-old *Pten^+/−^* mice were detectable by ultrasonogram, we analyzed wild type and *Pten^+/−^* mice with the Vevo770 high-resolution micro-imaging ultrasound system (Visualsonics). Consistently, *in vivo* imaging identified gallbladder polyps only in *Pten^+/−^* mice (Fig. 5). Histopathological analysis confirmed the presence of large tumors in the gallbladders of the *Pten^+/−^* mice, as indicated by the ultrasonogram (Fig. 5, right panel).

## DISCUSSION

Gallbladder cancer is a lethal malignancy with a mean survival rate of 6 months and a 5 year survival rate of 5% [26]. Lethality is mainly due to the fact that gallbladder cancer is generally asymptomatic during the early phases, with its diagnosis usually occurring at advanced stages [26]. Although possible risk factors such as ethnicity, gender, age, and life style might be associated with gallbladder cancer development, somatic genetic mutations impinging on specific oncosuppressors and oncogenes have been recently characterized in human gallbladder cancer [10-13]. Among them, mutations of the *PIK3CA* gene, known to render PI3K constitutively active, as well as loss of *PTEN* have been recently described [10, 12, 13]. However, whether deregulated activation of the PI3K/AKT pathway in human gallbladder carcinogenesis is a key factor in the onset of the disease, or alternatively a late event involved in the lethal progression of the tumor, is not yet understood [27]. Overall, our results demonstrate a clear role for PI3K/AKT pathway deregulation in gallbladder epithelial cell transformation as well as, importantly, in tumor growth. The papillomas observed in 12 month-old *Pten^+/−^* gallbladders are extensive lesions that frequently occupy 30%-50% of the entire organ, and are characterized by a high proliferative index, attributes that when associated with human gallbladder polyps generally indicate a high risk of malignant transformation. Importantly, the persistence of high levels of active Akt during onset and growth of these papillomas suggests the addiction of these lesions to the oncogenic PI3K/AKT signaling. These data strongly argue for the genetic stratification of advanced human gallbladder carcinomas based on PI3K or PTEN mutations and their treatment with small molecule pathway inhibitors. The *Pten^+/−^* mouse model may therefore represent an ideal platform for testing both pre- and co-clinically [28, 29], the efficacy of drugs designed to inhibit the PI3K pathway either as single drug agents or in combination with standard-of-care radio- or chemotherapy for the treatment of human gallbladder carcinoma.

## METHODS

### Ethics statement

All mouse work was done in accordance with our IACUC protocol.

### *Pten^+/−^*, *Pten^+/G129E^* mutant mice

*Pten^+/−^* and *Pten^+/G129E^* (C57BL/6) mice were generated as previously described [17, 21]. *Pten^+/−^*, *Pten^+/G129E^*, and wild type cohorts were generated by crossing *Pten^+/−^* or *Pten^+/G129E^* with wild type mice.

### Immunohistochemistry

For immunohistochemistry, gallbladders were fixed in 4% formaldehyde in PBS overnight, washed once with PBS, and dehydrated with 25%, 50%, and 70% ethanol. Gallbladders were embedded in paraffin, sectioned and stained with hematoxylin and eosin (H&E) in accordance with standard procedures. For immunohistochemistry (IHC), tissues were fixed in 4% formaldehyde and embedded in paraffin in accordance with standard procedures. Sections were stained for pSer473AKT (rabbit polyclonal 9271S, Cell Signaling Technology; 1:1:250), Ki67 (rabbit polyclonal, Novacastra; 1:400). Proliferative cells were identified by positive Ki-67 nuclear staining.

### Western blot

For Western blot, cell and tissue lysates were prepared with 150-RIPA buffer and protease and phosphatase inhibitor cocktails (Roche). The following antibodies were used for Western blotting: rabbit polyclonal anti-PTEN (138G6; 1:1000, Cell Signaling Technology), rabbit polyclonal anti-phospho-Akt(S473) (9271S; 1:1000, Cell Signaling Technology), rabbit polyclonal anti-Akt (9272S; 1:1000, Cell Signaling Technology), rabbit polyclonal anti-GAPDH (14C10; 1:6000, Cell Signaling Technology), rabbit polyclonal anti-p44/42 MAPK (Erk1/2) (9102S; 1:3000, Cell Signaling), rabbit polyclonal anti-Phospho-p44/42 MAPK (Erk1/2) (Thr202/Tyr204) (9101S; 1:3000, Cell Signaling).

### Imaging of gallbladder lesions

High-resolution ultrasound (US) imaging of normal and diseased mouse gallbladders was carried out using the Vevo 2100 System (Visual Sonics, Inc.). Briefly, mice were anesthetized with a 3% isoflurane/oxygen mixture and subsequently transferred to a platform heated to 39/40°C. Abdominal hair was removed by shaving and subsequent application of depilatory cream, followed by ample washing with sterile water to prevent irritation to the skin. Ultrasound gel was applied to the abdominal area of the mice and scanning performed with a with a 32–56 MHz Mircoscan™ transducer (MS-550S, Visual Sonics, Inc). Upon completion of imaging mice were returned to their cage and observed until fully recovered anesthesia.
